# Cross-cultural adaptation and reproducibility of the Brazilian-Portuguese version of the modified FRESNO Test to evaluate the competence in evidence based practice by physical therapists

**DOI:** 10.1590/bjpt-rbf.2014.0140

**Published:** 2016-01-19

**Authors:** Anderson M. Silva, Lucíola C. M. Costa, Maria L. Comper, Rosimeire S. Padula

**Affiliations:** 1 Programa de Mestrado e Doutorado em Fisioterapia, Universidade Cidade de São Paulo (UNICID), São Paulo, SP, Brazil; 2 Curso de Fisioterapia, Faculdades Integradas do Vale do Ribeira, Registro, SP, Brazil; 3 Curso de Fisioterapia, União Metropolitana de Ensino e Cultura, Itabuna, BA, Brazil

**Keywords:** evidence-based practice, modified Fresno scale, physical therapy, professional practice, professional education

## Abstract

**BACKGROUND:**

The *Modified Fresno Test* was developed to assess knowledge and skills of both physical therapy (PT) professionals and students to use evidence-based practice (EBP).

**OBJECTIVES:**

To translate the Modified Fresno Test into Brazilian-Portuguese and to evaluate the test's reproducibility.

**METHOD:**

The first step consisted of adapting the instrument into the Brazilian-Portuguese language. Then, a total of 57 participants, including PT students, PT professors and PT practitioners, completed the translated instrument. The responses from the participants were used to evaluate reproducibility of the translated instrument. Internal consistency was calculated using the Cronbach's alpha. Reliability was calculated using the intraclass correlation coefficient (ICC) for continuous variables, and the Kappa coefficient (K) for categorical variables. The agreement was assessed using the standard error of the measurement (SEM).

**RESULTS:**

The cross-cultural adaptation process was appropriate, providing an adequate Brazilian-Portuguese version of the instrument. The internal consistency was good (α=0.769). The reliability for inter- and intra-rater assessment were ICC=0.89 (95% CI 0.82 to 0.93); for evaluator 1 was ICC=0.85 (95% CI 0.57 to 0.93); and for evaluator 2 was ICC=0.98 (95% CI 0.97 to 0.99). The SEM was 13.04 points for inter-rater assessment, 12.57 points for rater 1 and 4.59 points for rater 2.

**CONCLUSION:**

The Brazilian-Portuguese language version of the *Modified Fresno Test* showed satisfactory results in terms of reproducibility. The *Modified Fresno Test* will allow physical therapy professionals and students to be evaluated on the use of understanding EBP.

## Introduction

Evidence-Based Practice (EBP) can be defined as the use of relevant scientific evidence to guide clinical decision making and to optimize health outcomes of patients^1^. Therefore, EBP incorporates knowledge related to clinical research of high quality, professional knowledge, and patient's preferences^1^^-^ 4. This approach has been increasingly used by physical therapists. In order to be effective and applicable in clinical practice, EBP should be able to^3^^,^ 5: formulate a clinical question; drive effective searches of databases; critically assess the methodological quality of study findings; and provide the evidence for therapeutic decisions^2^^,^ 6 ^-^ 10. Any difficulty in performing these steps could be a barrier to the adoption of EBP^5^. To that end, adequate training^11^^-^ 13 and the constant evaluation of knowledge and skills acquired by the professionals during their training using reliable instruments are fundamental^14^^,^ 15.

The concept of competence is broad^16^, and although there is no single definition, the consensus is that integration of personal attributes to the actions carried out in specific contexts, aimed at achieving results, are key aspects^17^. In the physical therapy context, competence indicates the capacity to manipulate cognitive resources that add knowledge, information, skills, and, above all, intelligence, to effectively and with relevance, solve a number of situations in clinical practice^18^^,^ 19. The term could also incorporate the knowledge domain^2^, ability to work in teams, and to be effective in verbal and written communication^2^^,^ 17 ^,^ 20.

Although there are a number of instruments^12^ that are used to assess the EBP in health professionals have been identified, few have had their validity and reliability tested or have failed to evaluate EBP in its entirety^21^. The Fresno Test^1^^,^ 2 ^,^ 14, originally developed to assess the ability of medical professionals to evaluate medical literature, is the only instrument that evaluates all stages of EBP^2^^,^ 12. It was later adapted to evaluate other health professionals such as nurses^18^, occupational therapists^22^ and physical therapists^7^^,^ 18. This Modified Fresno Test^7^ was developed to assess the knowledge and skills of physical therapy professionals and students to use EBP principles in their daily professional practices & classes. The instrument identifies the whether the individual has the academic and professional knowledge necessary to be able to use high methodological quality evidence for clinical decision-making.

In the absence of instruments that assess the knowledge and skills of Brazilian PT students and professionals to use EBP, the need for a cross-cultural adaptation of a suitable test instrument to be used in the Brazilian-Portuguese language becomes evident. Thus, this study aimed to translate and cross-culturally adapt the Modified Fresno Test for physical therapists, and to evaluate the reproducibility of the instrument in the Brazilian-Portuguese version.

## Method

### The Modified Fresno Test instrument

The Modified Fresno Test is a self-explanatory tool that evaluates competencies and skills of professionals and students to use EBP^7^. The instrument is composed of an initial text with instructions for completion and two clinical scenarios. Each participant must choose one of the scenarios provided and answer 13 questions pertaining to the chosen scenario. Questions 9 and 10 require mathematical calculations. The questionnaire must be answered within the maximum permitted time of sixty minutes.

The total score is calculated from the partial score of each question which has 4 possible answers (a to d) to choose from. For example, question 2 asks the participant to name the research databases selected to answer the clinical question, which has the following items listed: (a) different resources; (b) convenient resources; (c) clinical relevant resources; and (d) valid resources. The response or answer for each item is then scored using one of five classification categories as follows: (1) not clear; (2) limited; (3) minimum; (4) strong; and (5) excellent^7^. The sum of scores for each criterion results in a score per question that ranges from 0 to 24 points: Q1 to Q7 allow a maximum score of 24 points; Q8 and Q10 a maximum of 16 points; Q9 allows 12 points; and Q11 to Q13 a maximum of 4 points. The total test score is the sum of the points from all questions, ranging from 0 to 224 points^7^ (Table 1). After the participant responded to all of the questions, two trained raters scored the responses.

**Table 1.** Modified Fresno Test for physical therapists - Brazilian-Portuguese Edition: Percentage and scoring of the questions according to the steps of Evidence Based Practice.

**Step/Action**	**Questions - Q**	**Scores**	**% and total score**
Step 1: Elaboration of the question	Q1 - Formulate a clinical question	Q1 - (0 to 24)	11% (24)
Step 2: Search for the best available evidence	Q2 - Information Sources Q4 - Search (search strategy)	Q2 - (0 to 24) Q4 - (0 to 24)	21% (48)
Step 3a: Critical evaluation (qualitative evidence)	Q3 - Study design Q5 - Relevance Q6 - Internal Validity Q7 - Magnitude and significance Q12 - Best study design (diagnosis) Q13 - Best study design (prognosis)	Q3 - (0 to 24) Q5 - (0 to 24) Q6 - (0 to 24) Q7 - (0 to 24) Q12 - (0 or 4) Q13 - (0 or 4)	46% (104)
Step 3b: Critical evaluation (quantitative evidence)	Q9 - Sensitivity, positive predictive value and positive likelihood ratio Q10 - Absolute risk reduction, relative risk, NNT, and p-value Q11 - Confidence Interval	Q9 - (0 to 12) Q10 - (0 to 16) Q11 - (0 or 4)	14% (32)
Step 4: Implementation of evidence into clinical practice	Q8 - Questioning the patient/family	Q8 - (0 to 16)	7% (16)
		Total (0 to 224)	Total 100% (224)

Q: Question; NNT: Number needed to treat.

### Cross-cultural adaptation of the Modified Fresno Test

The first step was to cross-culturally adapt the Modified Fresno Test^7^ to the Brazilian-Portuguese language. To translate and adapt the instrument, the guidelines for translation and adaptation of health care questionnaires, proposed by Beaton et al.^19^, were adopted^19^. Thus, the following steps were conducted: translation, synthesis of the translation, back-translation into English, summary of the back-translation, evaluation of the new document by an expert committee followed by a pre-test.

The resulting version of the translation process was pre-tested using 30 physical therapists who had 1 to 10 years (mean=5.7; SD=3.1) experience who may or may not have had experience using EBP principles. The participants were 22 females and 8 males, between the ages of 25 and 36 years, who had graduated from public or private universities that were not involved in the first part of the study. Of those, 21 participants received the questionnaire in digital format and 9 in print format. Each participant was asked to read and answer the questionnaire and at the end of each question, there was a blank field with the text "*here, describe your suggestions and/or difficulty in answering this question*", so that they could report any difficulties encountered when understanding the questions. Once completed, the questionnaires were scored by two investigators with experience in EBP practice, working in the areas of musculoskeletal and cardiorespiratory physical therapy for 7 years.

### Measurement properties

This phase tested the measurement properties of the Modified Fresno Test for the Brazilian-Portuguese version, following the guidelines proposed by Mokkink et al.^23^ for testing internal consistency and intra- and inter-rater reliability, and the guidelines of Terwee et al.^24^ to test the compliance of the instrument.

Reliability measures the accuracy of an instrument to provide the same answer in repeated measurements, representing the relative error of the measurement. It can be measured when the instrument is used more than once by the same examiner (intra-rater reliability) and when the instrument is used to evaluate the same condition by different raters (inter-rater reliability)^23^^,^ 25 ^,^ 26. The correlation represents the degree to which the repeated analysis of the same individual provided similar responses^24^^,^ 25.

The statistical tests adopted for the reliability analysis were the same ones used with the original instrument^7^. The Cronbach's alpha (α) was used to assess the internal consistency of the instrument (ranging between 0.70 and 0.95), the intraclass correlation coefficient type_2,1_ (ICC_2,1_) was used to test the reliability of the numeric or quantitative variables in continuous scales^27^, and the Kappa coefficient (K) was used for the categorical variables. The ICC was classified according to Fleiss^27^ as poor (<0.4), good (0.4 to 0.75) and excellent reliability (>0.75). The Kappa was classified according to Landis and Koch^28^ as reliability being almost perfect (>0.81), substantial (0.61 to 0.80), moderate (0.41 to 0.60), low (0.21 to 0.40) and very low (0.00 to 0 20). The reliability tests were performed with a 95% confidence interval.

The agreement between the assessors was assessed using the standard measurement error (SME), which reflected the error of the instrument, expressed by the standard deviation of the differences of the test and re-test, divided by the square root of 2^24^. The relationship between the SME and the total score of the questionnaire was interpreted as proposed by Ostelo et al.^29^ who considered values ≤5% as very good, >5% and ≤10% as good, >10% and ≤20% as doubtful and >20% as negative. All data collected were transferred to the software Statistical Package for Social Sciences (SPSS) version 17.0 for analysis.

### Procedures

This study was approved by the Ethics Committee of the Universidade Cidade de São Paulo (UNICID), São Paulo, SP, Brazil, under the Research Protocol number 13696713/2012 and authorized by the authors who developed the original instrument. All participants signed an informed consent form.

The Modified Fresno Test was translated into Brazilian-Portuguese by two bilingual (Portuguese and English) translators, both with over 15 years of linguistics training, whose first language was Brazilian-Portuguese. Translator one had 11 years experience in higher education, was knowledgeable in health terminology and was aware of the instrument goals, while translator two had 9 years experience in higher education, but no knowledge of how EPB was used, or experience in the health area. Next, the translated versions were compared and analyzed and a consensual approach by the 2 translators to resolve any differences of interpretation and translation was conducted. Thus, the two independent translations (T_1_ and T_2_) were created and then synthesized into a single consensual translation, termed T_12_.

The T_12_ version was then back translated into English by two different independent translators (back-translator 1 and back-translator 2). These translators had backgrounds in physical therapy and were knowledgeable in the EBP methodology and were fluent in Portuguese and English, with the latter being their mother tongue. In addition, these back- translators, although aware of EBP methodology, were unaware of the Modified Fresno Test. The results of the two back-translations (RT_1_ and RT_2_) were synthesized in a consensual manner similar to the original translations resulting in a single consensual translation called RT_12_.

Finally, a Committee of Experts, consisting of methodologists, health professionals and professional translators (2 translators, 2 back-translators, 6 physical therapists with teaching experience in EBP), evaluated and consolidated all versions (R12 & T12). The committee compared the translations and discussed the semantic, structural, idiomatic and conceptual properties from the Brazilian-Portuguese version and developed a pre-final version. After conducting the pre-test, barely any changes were made to the final version.

To test the final version of the instrument, a convenience sample of 159 physical therapists were invited to participate. The sample size was estimated as proposed by the guidelines for reliability tests^24^^,^ 30 ^,^ 31. The sample consisted of: (1) physical therapy professors of public and private institutions involved in the clinical practice, (2) students enrolled on the third year of their PT program and (3) physical therapists not connected to higher education institutions, independent of the their familiarity with EBP.

The volunteers were instructed to use a notebook and a calculator to assist in answering all of the questions. They were asked to complete the task within the stipulated time (60 minutes) even though the original study had not justified the time parameters. Data collection was conducted from April to September 2013.

The evaluators received a single training, divided into three steps of one hour each. The first hour was an orientation about the criteria and scoring of the questions. The second hour was a pilot test in which each evaluator scored one sample test. The third hour was for analysis and discussion of the score results. The evaluators were blinded to avoid identification of the participants and the score of the instrument during the test and re-test.

After the training, the evaluators received the questionnaires answered by 57 participants and scored the answers to obtain the partial and total test scores of the Modified Fresno Test. Inter- and intra-rater reliability tests and inter-rater agreement of the partial and full scores of the instrument were carried out using a test/retest design with an interval of seven days between scoring sessions. The test/retest was performed by two independent evaluators who did not participate in the previous stages.

## Results

Of the 159 individuals invited to participate in the study, only 32% (n=57) returned the questionnaires. Of those, 36 were PT professionals (13 were also PT teachers in the clinic) with an average working experience time of 6.6 years, (SD-3.8) and 21 were PT academics (last year program). Thirty-seven answered the questionnaire in print and 20 in the digital version. The 95 participants who did not return the questionnaires were asked about their reasons for not returning the test information. Some claimed lack of knowledge of the topic and seven expressed lack of interest in the research. None of them reported difficulty in understanding the questions, but reported a lack of knowledge for answering the topics covered. The percentage of missing items per question for the respondents of the questionnaire (n=57) is shown in Table 2. The evaluators who scored the answers of the test/re-test did not report any problems.

**Table 2.** Measurement properties of the Modified Fresno Test - Brazilian-Portuguese version per item and the total sum of the questions.

**Questions**	**Inter-rater**	**Intra-rater 1**	**Intra-rater 2**			
	**(n=57)**	**(n=57)**	**(n=57)**	**(n=57)**	**(n=57)**	**(n=57)**
	**ICC_2, **1**_ (95% CI)**	**Agreement - (SEM)**	**ICC_2, **1**_ (95% CI)**	**Agreement - (SEM)**	**ICC_2, **1**_ (95% CI)**	**Agreement - (SEM)**
Q1	0.75 (0.60-0.84)	3.40	0.82 (0.69-0.90)	2.80	0.98 (0.97-0.99)	0.76
Q2	0.52 (0.30-0.68)	3.81	0.47 (0.20-0.67)	4.06	0.96 (0.94-0.97)	1.07
Q3	0.89 (0.82-0.93)	2.51	0.86 (0.78-0.91)	3.01	0.97 (0.95-0.98)	1.22
Q4	0.75 (0.61-0.84)	3.11	0.80 (0.68-0.88)	2.64	0.97 (0.95-0.98)	1.05
Q5	0.66 (0.48-0.78)	2.97	0.49 (0.22-0.68)	3.70	0.91 (0.85-0.94)	4.06
Q6	0.68 (0.51-0.79)	4.44	0.79 (0.67-0.87)	3.60	0.94 (0.90-0.96)	1.84
Q7	0.58 (0.38-0.73)	4.59	0.67 (0.50-0.79)	4.10	0.94 (0.90-0.96)	4.85
Q8	0.65 (0.47-0.78)	2.53	0.64 (0.31-0.81)	2.52	0.96 (0.93-0.97)	0.83
Q9	0.90 (0.84-0.94)	1.11	0.97 (0.95-0.98)	0.65	0.99 (0.98-0.99)	0.30
Q10	0.94 (0.90-0.96)	0.94	0.94 (0.90-0.96)	0.95	0.99 (0.99-0.99)	1.96
Q11	0.92 (0.87-0.95)	0.37	0.92 (0.87-0.95)	NC	1.00 (1.00-1.00)	0.37
Q12	0.75 (0.60-0.84)	0.97	0.89 (0.83-0.93)	0.65	1.00 (1.00-1.00)	0.97
Q13	0.93 (0.88-0.95)	0.53	0.93 (0.88-0.95)	0.53	1.00 (1.00-1.00)	NC
Total	0.89 (0.82-0.93)	13.04	0.85 (0.57-0.93)	12.57	0.98 (0.97-0.99)	4.59

Missing data (n=57) - Q9=7 (12.3%); Q10=5 (8.7%); Q11=4 (7.0%); Q13=1(1.8%). NC: The statistical program did not allow the calculation of these items.

### Cross-cultural adaptation of the Modified Fresno Test for Brazilian-Portuguese

When the original version was compared with the versions resulting from the translations (T_1_ and T_2_) and back-translations (RT_1_ and RT_2_), no significant differences were found for the content and meaning. Grammatical adjustments were conducted for different words but the same meaning was maintained between the two versions.

In the pre-test, participants reported no problems understanding the terms and questions. However, six of the 30 physical therapists who answered the instrument (two answered in print version and four in digital) reported difficulties understanding the instrument's instructions. Through these results, adjustments were made to facilitate understanding and the text was forwarded to the Committee of Experts, resulting in the final Modified Fresno Test - Brazilian-Portuguese version (Appendix 1 and 2).

### Evaluation of reliability

The Brazilian-Portuguese version of the test showed good internal consistency (α=0.769). For evaluator 1, the ICC ranged from 0.47 to 0.97. The agreement of the SEM varied from 0.53 to 4.10 points. Evaluator 1 showed excellent reliability, except for questions 2 and 5, which showed an ICC of 0.47 and 0.49, respectively, indicating moderate reliability. In relation to questions 11, 12 and 13, the Kappa coefficient obtained was excellent and ranged from 0.89 to 0.93.

For evaluator 2, reliability was found to be higher compared to values reported for evaluator 1. The ICC ranged from 0.98 to 0.99 and the SEM agreement ranged from 0.30 to 4.85 points. These results represented excellent reliability for all questions tested in the instrument. Questions 11, 12 and 13 showed excellent reliability with a Kappa index of 1.0. The inter-rater reliability presented an ICC that ranged from 0.52 to 0.94 and an agreement by the SEM that ranged from 0.37 to 4.59 points.

The inter-rater reliability between the 2 evaluators proved to be excellent except for question 2, which resulted in an ICC of 0.52 indicating moderate reliability. Questions 11, 12 and 13 showed good to excellent reliability, with a Kappa index ranging from 0.75 to 0.93.

For the total score of the instrument, the intra-rater reliability was excellent for both evaluators. Evaluator 1 had an ICC of 0.85 (0.57 to 0.93) and evaluator 2, an ICC of 0.98 (0.97 to 0.99). The inter-rater reliability was excellent with an ICC of 0.89 (from 0.82 to 0.93). The agreement by the SEM was 13.04 points for the inter-rater assessment, 12.57 points for evaluator 1 and 4.59 points for evaluator 2.

## Discussion

The purpose of this study was to adapt and test the reliability properties and agreement of a Brazilian-Portuguese version of the Modified Fresno Test, an instrument, that to date, is considered to be the most complete and appropriate test for evaluating PT professionals and students knowledge and skills on the use of EBP^12^.

The adaptation process to the Brazilian-Portuguese version of the Modified Fresno Test followed the steps indicated by the available literature^19^. The steps were completed without difficulty and adjustments were made to the content and meaning of the text. Although most of the participants failed to complete the final version of the questionnaire, the reasons were unrelated to the difficulties with the topic^10^ or in understanding the questions. Therefore, it was not considered a barrier in the instrument adaptation process, since different levels of skills and competence in relation to EBP was expected, as mentioned by the authors who developed the instrument^7^.

The question with the highest amount of missing data was Q9, which required statistical knowledge, followed by Q10, Q11 and Q13. The omission of EPB learning during professional training, the difficulty in dealing with statistics and conducting the databases search were the reasons for participants dropping out of this study^32^. It is possible that those reports were the major obstacles facing physical therapists, independent of their professional time in the process of adopting EBP. Difficulty in the searching process, interpretation and in translating the evidence into clinical practice is related to the competencies and skills of a professional; however, younger professionals have shown a more positive attitude to the adoption of EBP^33^. Nevertheless, other obstacles have been pointed out by other studies in the adoption of EBP, such as limited access to databases and complete manuscripts, language issues, and the time available to learn the topic^5^^,^ 6 ^,^ 33 ^-^ 35.

The original version of the instrument lacked classification categories for the total score of the questions, but the higher the values, the better the participants were in answering each question or a group of questions. The results of the reliability tests for the total score of the modified instrument, obtained by using the Brazilian-Portuguese version, were considered excellent for the intra- and inter-rater assessment. The study, which validated the modified instrument for physical therapists, presented good internal consistency (α=0.769), similar to the values obtained with the original unmodified instrument (0.780). In addition, the modified English version of the instrument presented an excellent reliability inter- and intra-observer of 0.92 (95% CI 0.88 to 0.94), for evaluator 1 an ICC of 0.96 (95% CI .91-.98), and for evaluator 2 of 0.96 (95% CI from 0.91 to 0.98)^7^.

The scores of the partial questions of the adapted instrument showed moderate to excellent results for intra- and inter-rater reliability of the evaluators. These results were similar to the original English version of the modified instrument which presented moderate to excellent intra-observer reliability for the evaluators for all questions (ICC ranging from 0.62-1.0) and moderate to excellent inter-rater reliability (ICC ranging from 0.61 to 0.99) for all questions of the instrument; however, differences between the two versions were observed for questions 2, 5 and 8.

Question 8 evaluated the ability of the participants to obtain information about the patient's perspective, which presented unsatisfactory reliability (ICC_2,1_=0.47) in the original version and good reliability (ICC_2,1_=0.65) in the Brazilian-Portuguese version for the inter-rater analysis. Question 2 evaluated the participant's knowledge and ability to search the database, and presented a moderate reliability for evaluator 1 and for the inter-rater assessment with the Brazilian-Portuguese version. In the original English version, reliability values were excellent for this question (ICC_2,1_=0.83), as well as for evaluator 1 and for the inter-rater assessment (ICC_2,1_=0.90). Question 5 referred to the participant's ability to determine the clinical relevance of the identified studies, and showed lower reliability values in the Brazilian-Portuguese version than in the original version for evaluator 1 (ICC_2,1_=0.49). The score differences between the two versions for this question might be related to the level of knowledge acquired during their professional training^15^ of EBP between participants.

The values of the inter-rater reliability and intra-rater SEM for the Brazilian-Portuguese version showed little variability in the intra- and inter-rater scores, considering that the total instrument score ranged from 0 to 224 points. These results could not be compared with the original modified version because this property was not assessed in the original version^7^. Upon completion of the translation phases and testing of the measurement properties, the Brazilian-Portuguese version of the Modified Fresno Test for physical therapists proved to be similar in terms of the language and clearly understood by the Brazilian population (Appendix 1*). In addition, it could be a useful tool for researchers and educational institutions in assessing the ability of Brazilian professionals to adopt EBP. According to the strategy of using the Fresno Test for medical doctors and the Modified Fresno Test for physical therapists, cited in the respective studies^7^^,^ 14, the authors believe that this instrument is best used for continuous evaluation, which involves a constant process of assessments and reassessments to ensure people understand EBP principles and if they do not, have taken the time or are given the opportunity to develop skills in EBP.

The difficulty in obtaining a greater number of participants to answer the questionnaire during the test and after 24 hours, ensuring that there was no time to supplement the information from websites or books, was a limitation of this study because only 11 individuals returned the questionnaire on time. Therefore, the present study only tested the reliability and agreement of the evaluators of the Modified Fresno Test version adapted to Brazilian-Portuguese. Therefore, the authors recommend the development of new studies in order to test other properties of the instrument, such as construct validity and responsiveness of the Brazilian-Portuguese version. Further studies are needed to continually assess the new learning methods and the use of scientific evidence in therapeutic decisions during students professional training, because these methods are fundamental prerequisite for the adoption of EBP principles.

## Conclusion

The Modified Fresno Test for physical therapists revealed satisfactory results in the adaptation process. The Brazilian-Portuguese version of the instrument showed good internal consistency, excellent reliability and low variability in the intra-agreement test and for inter-evaluators. There are other properties that should be tested. However, the steps taken so far may contribute to the development of studies aimed at assessing comprehensively the knowledge and skills of physical therapy professionals and students in the use of EBP in their clinical practice.

## References

[1] Herbert R, Jamtvedt G, Mead J, Hagen KB (2011). Practical evidence-based physiotherapy.

[2] Ilic D (2009). Assessing competency in evidence based practice: strengths and
limitations of current tools in practice. BMC Med Educ.

[3] Herbert R, Jamtvedt G, Mead J, Hagen KB (2005). Practical evidence-based physiotherapy.

[4] Shiwa SR, Costa LOP, Moser ADL, Aguiar IC, Oliveira LVF (2011). PEDro: a base de dados de evidências em fisioterapia. Fisioter Mov.

[5] Jette DU, Bacon K, Batty C, Carlson M, Ferland A, Hemingway RD (2003). Evidence-based practice: beliefs, attitudes, knowledge, and behaviors
of physical therapists. Phys Ther.

[6] Connolly BH, Lupinnaci NS, Bush AJ (2001). Changes in attitudes and perceptions about research in physical
therapy among professional physical therapist students and new
graduates. Phys Ther.

[7] Tilson JK (2010). Validation of the modified Fresno Test: assessing physical therapists'
evidence based practice knowledge and skills. BMC Med Educ.

[8] Glasziou P, Del Mar C, Salisbury J (2010). Prática clínica baseada em evidências: livro de exercícios.

[9] Claridge JA, Fabian TC (2005). History and development of evidence-based medicine. World J Surg.

[10] Silva TM, Costa LCM, Costa LOP (2015). Evidence-based practice: a survey regarding behavior, knowledge,
skills, resources, opinions and perceived barriers of Brazilian physical
therapists from São Paulo state. Braz J Phys Ther.

[11] Marques AP, Peccin MS (2005). Pesquisa em fisioterapia: a prática baseada em evidências e modelos de
estudos. Fisioter Pesqui.

[12] Shaneyfelt T, Baum KD, Bell D, Feldstein D, Houston TK, Kaatz S (2006). Instruments for evaluating education in evidence-based practice: a
systematic review. JAMA.

[13] Coury H, Vilella I (2009). Profile of the Brazilian physical therapy researcher. Rev Bras Fisioter.

[14] Ramos KD, Schafer S, Tracz SM (2003). Validation of the Fresno test of competence in evidence based
medicine. BMJ.

[15] Sackett DL, Rosenberg WM, Gray JA, Haynes RB, Richardson WS (1996). Evidence based medicine: what it is and what it isn't. BMJ.

[16] Domingues RCL, Amaral E, Bicudo-Zeferino AM (2009). Global rating: a method for assessing clinical
competence. Rev Bras Educ Med.

[17] Lima VV (2005). Competência: distintas abordagens e implicações na formação de
profissionais de saúde. Rev Interface.

[18] Morris J, Maynard V (2009). The feasibility of introducing an evidence based practice cycle into a
clinical area: an evaluation of process and outcome. Nurse Educ Pract.

[19] Beaton D, Bombardier C, Guillemin F, Ferraz MB (2000). Guidelines for the process of cross-cultural adaptation of self-report
measures. Spine (Phila Pa 1976).

[20] Brasil. Conselho Nacional de Educação - CNE (2002). Resolução CNE/CES nº 4, de 19 de fevereiro de 2002. Institui Diretrizes
Curriculares Nacionais do Curso de Graduação em Fisioterapia.

[21] Kogan JR, Holmboe ES, Hauer KE (2009). Tools for direct observation and assessment of clinical skills of
medical trainees: a systematic review. JAMA.

[22] McCluskey A, Bishop B (2009). The adapted Fresno test of competence in evidence-based
practice. J Contin Educ Health Prof.

[23] Mokkink LB, Terwee CB, Patrick DL, Alonso J, Stratford PW, Knol DL (2010). The COSMIN study reached international consensus on taxonomy,
terminology, and definitions of measurement properties for health-related
patient-reported outcomes. J Clin Epidemiol.

[24] Terwee CB, Bot SD, de Boer MR, van der Windt DA, Knol DL, Dekker J (2007). Quality criteria were proposed for measurement properties of health
status questionnaires. J Clin Epidemiol.

[25] Maher CG, Latimer J, Costa LOP (2007). The relevance of cross-cultural adaptation and clinimetrics for
physical therapy instruments. Rev Bras Fisioter.

[26] Puga VOO, Lopes AD, Costa LOP (2012). Assessment of cross-cultural adaptations and measurement properties of
self-report outcome measures relevant to shoulder disability in portuguese: a
systematic review. Rev Bras Fisioter.

[27] Fleiss JL (1986). The design and analysis of clinical experiments.

[28] Landis JR, Koch GG (1977). The measurement of observer agreement for categorical
data. Biometrics.

[29] Ostelo RWJG, de Vet HCW, Knol DL, van den Brandt PA (2004). 24-item Roland-Morris Disability Questionnaire was preferred out of
six functional status questionnaires for post-lumbar disc surgery. J Clin Epidemiol.

[30] Moriguchi CS, Alem MER, Veldhoven M, Coury HJCG (2010). Cultural adaptation and psychometric properties of Brazilian need for
recovery scale. Rev Saude Publica.

[31] Argimon-Pallas JM, Flores-Mateo G, Jimenez-Villa J, Pujol-Ribera E (2010). Psychometric properties of a test in evidence based practice: the
spanish version of the Fresno test. BMC Med Educ.

[32] Silva TM, Costa LCM, Garcia AN, Costa LOP (2015). What do physical therapists think about evidence-based practice? A
systematic review. Man Ther.

[33] Queiroz PS, Santos MJD (2013). Facilidades e habilidades do fisioterapeuta na procura, interpretação
e aplicação do conhecimento científico na prática clínica: um estudo
piloto. Fisioter Mov.

[34] Akinbo S, Odebiyi D, Okunola T, Aderoba O (2008). Evidence-based practice: knowledge, attitudes and beliefs of
physiotherapists in Nigeria. Int J Med Inform.

[35] Schreiber J, Stern P (2005). A review of the literature on evidence-based practice in physical
therapy. Internet J Allied Health Sci Pract.

